# Perspectives of older people about contingency planning for falls in the community: A qualitative meta-synthesis

**DOI:** 10.1371/journal.pone.0177510

**Published:** 2017-05-31

**Authors:** Kimberly Charlton, Carolyn M. Murray, Saravana Kumar

**Affiliations:** 1 Domiciliary Care, Department of Communities and Social Inclusion, South Australian Government, Northfield, South Australia, Australia; 2 School of Health Sciences, University of South Australia, Sansom Institute for Health Research, Adelaide, South Australia, Australia; Cardiff University, UNITED KINGDOM

## Abstract

**Objective:**

Despite consistent evidence for the positive impact of contingency planning for falls in older people, implementation of plans often fail. This is likely due to lack of recognition and knowledge about perspectives of older people about contingency planning. The objective of this research was to explore the perspectives of older people living in the community about use of contingency planning for getting help quickly after a fall.

**Method:**

A systematic literature search seeking qualitative research was conducted in April 2014, with no limit placed on date of publication. Medline, EMBASE, Ageline, CINAHL, HealthSource- Nursing/Academic Edition, AMED and Psych INFO databases were searched. Three main concepts were explored and linked using Boolean operators; older people, falls and contingency planning. The search was updated until February 2016 with no new articles found. After removal of duplicates, 562 articles were assessed against inclusion and exclusion criteria resulting in six studies for the meta-synthesis. These studies were critically appraised using the McMaster critical appraisal tool. Bespoke data extraction sheets were developed and a meta-synthesis approach was adopted to extract and synthesise findings.

**Findings:**

Three themes of ‘a mix of attitudes’, ‘careful deliberations’ and ‘a source of anxiety’ were established. Perspectives of older people were on a continuum between regarding contingency plans as necessary and not necessary. Levels of engagement with the contingency planning process seemed associated with acceptance of their risk of falling and their familiarity with available contingency planning strategies.

**Conclusion:**

Avoiding a long lie on the floor following a fall is imperative for older people in the community but there is a lack of knowledge about contingency planning for falls. This meta-synthesis provides new insights into this area of health service delivery and highlights that implementation of plans needs to be directed by the older people rather than the health professionals.

## Introduction

The proportion of older people is growing and there is an associated expectation that older people living in the community who are at risk of falls will also increase [1]. Falls are the leading cause of injury-related hospital admissions for people over 65 in Australia, with an estimated 30 to 40 percent of community dwelling older people having a fall every year [2]. Older people living at home are often unable to get up following a fall, with one study finding that 30% of older people who fell remained on the floor for over an hour [3]. Falls resulting in a person remaining on the floor for one hour or more (a long lie) may result in complications of muscle weakness, development of pressure areas, pneumonia, dehydration, missing medicines and hypothermia [4–6]. Following a long lie on the floor, a self-imposed reduction in mobility and social isolation can impact upon quality of life and place increased demands upon carers [6, 7].

Contingency planning for a fall has potential to reduce both medical and functional complications associated with falling. Contingency planning can enable older people in the community to access help quickly and can be described as a plan of action to be taken in anticipation of a foreseeable emergency that may involve the risk of serious harm [8]. There are a range of recognised strategies which constitute contingency planning for falls, including; personal alarm response systems (PERS), automated fall detectors (AFD), pagers, mobile phones, Global Positioning Systems (GPS), phone checking services, and neighbour/family alert or checking systems [2].

Existing research on contingency planning for falls has been focused largely on the consumer perspective of using technology for “managing the outcome of a fall after it has occurred” ([9], p. 203), the utility of PERS from a consumer perspective [1, 10], and perspectives about use of home monitoring technologies, including automated fall detectors [11, 12]. These AFD systems have sensors which detect body position and speed of movement. If the system detects a fall, it will automatically activate to get assistance. Research has found that older participants were accepting of automated wearable fall detectors [13] but there were associated feelings of increased vulnerability and worry about impact on identity [14] invasion of privacy and concerns about cost, appearance and maintenance of the technology [7].

Survey findings have suggested that older people fear accidentally activating a PERS or do not recognise their need for one [1, 10, 15] with its reported usage following a fall as low as 12% [11]. To inform service delivery, it is necessary to know more about why the usage of PERS is so low following a fall and not assume that this occurs due to a reluctance to use technology [9]. It is also important to know more about what older people prefer as contingency planning options for a fall and how they make decisions about this in order to promote the uptake of contingency planning. One method for understanding more about perspectives of contingency planning for a fall is by bringing together findings from existing qualitative research in a meta-synthesis [16]. This method will provide collective and enhanced understanding about some of the potential barriers and enablers of contingency planning and the utility and desirability of different options. Therefore, the aim of this review was to explore perspectives of older people living in the community about use of contingency planning for getting help quickly after a fall.

## Methods

### Search strategy

The following databases were searched in April 2014 (and updated in February 2016 to capture any additional publications since the conduct of the original search) with no limit placed on date of publication. Medline, EMBASE, Ageline, CINAHL, HealthSource- Nursing/Academic Edition, AMED, Psych INFO. In order to avoid publication bias, grey literature was also sought through state and national government websites and reference lists were reviewed (pearling). In the search process, three main concepts were explored; older people, falls and contingency planning and linked using Boolean operators (AND and OR). Table 1 provides an overview of all search terms under each main concept. MESH descriptors were utilised to increase search functions for all main concepts.

**Table 1:** Key concepts and search terms.

	Key Concepts	Search Terms
Combined using AND	Older Person	age*OR elderly OR old*OR frail*OR senior*
	Fall	fall* OR stumble OR tumble OR long lie* OR trip* OR slip*
	Contingency Planning	contingency plan* OR plan*, OR emergency plan* OR management plan* OR emergenc* OR safety plan* OR personal safety plan* OR back up plan* OR emergency response* OR telephone monitor* OR phone monitor* OR home visit* OR check visit* OR check call* OR telecross* OR Red Cross fall* OR detection OR automated fall detect* OR fall monitor* OR personal alarms OR autodiallers OR emergency response systems OR emergency pendants OR call direct or personal alert system*

### Inclusion and exclusion criteria

As this was a qualitative meta-synthesis, primary qualitative research was sought that explored individual experiences and reported these using thick description. Thick description means that the findings were interpreted and explained by the authors rather than providing simple descriptive summaries of data [17]. Participants who were over 65 were sought because one in three of this population fall each year and the risk of falling increases with age [18]. Research that was not in English or was not about perspectives of older people about getting help following a fall was excluded.

### Critical appraisal

The quality of the studies was assessed using the indicators of credibility, transferability, dependability and confirmability as defined in Liamputtong [19] and guided by the McMaster critical appraisal tool for qualitative research [20]. This tool was chosen because it is freely available, is widely used to appraise qualitative research and the authors had previous experience in using this tool. Appraisal of credibility was based on declaration of author biases or preconceptions, attempts to incorporate triangulation, collection of data over a prolonged period or undertaking member checking to verify data interpretation [19]. Appraisal of transferability was based on the applicability of the research to other contexts and whether there was adequate detail given about the sample [19]. Appraisal of dependability was based on clear explanation of the methods of data collection, analysis and interpretation to enable replication [19]. Appraisal of confirmability was dependent on evidence of strategies to limit bias and maintain neutrality of author interpretation [19]. To allow for consistency of the interpretation of study quality, independent appraisals of the studies were completed by two authors and then compared (KC and CM).

### Data extraction and synthesis

A meta-ethnographic approach, developed by Nobilt and Hare [21] was used for extraction and synthesis of findings from the chosen studies. This approach was well established and rigorous and included three levels of analysis [22]. To complete the first level of analysis, the first author (KC) read the studies multiple times and made notes about key findings. Each study was allocated a colour and the findings (including themes and quotes) were extracted into an electronic file. The findings from different studies were grouped together into concepts to arrive at the second level of analysis known as reciprocal translation [22]. The third level of analysis aimed to reduce the data into a manageable synthesis organised into broad themes. This process involved systematic comparison and interpretation of concepts to arrive at a new interpretation of the research when combined [22]. This process resulted in three overarching themes which were reviewed by another author (CM) to enhance rigour and minimise potential biases.

## Findings

### Search outcomes

**Fig 1:**
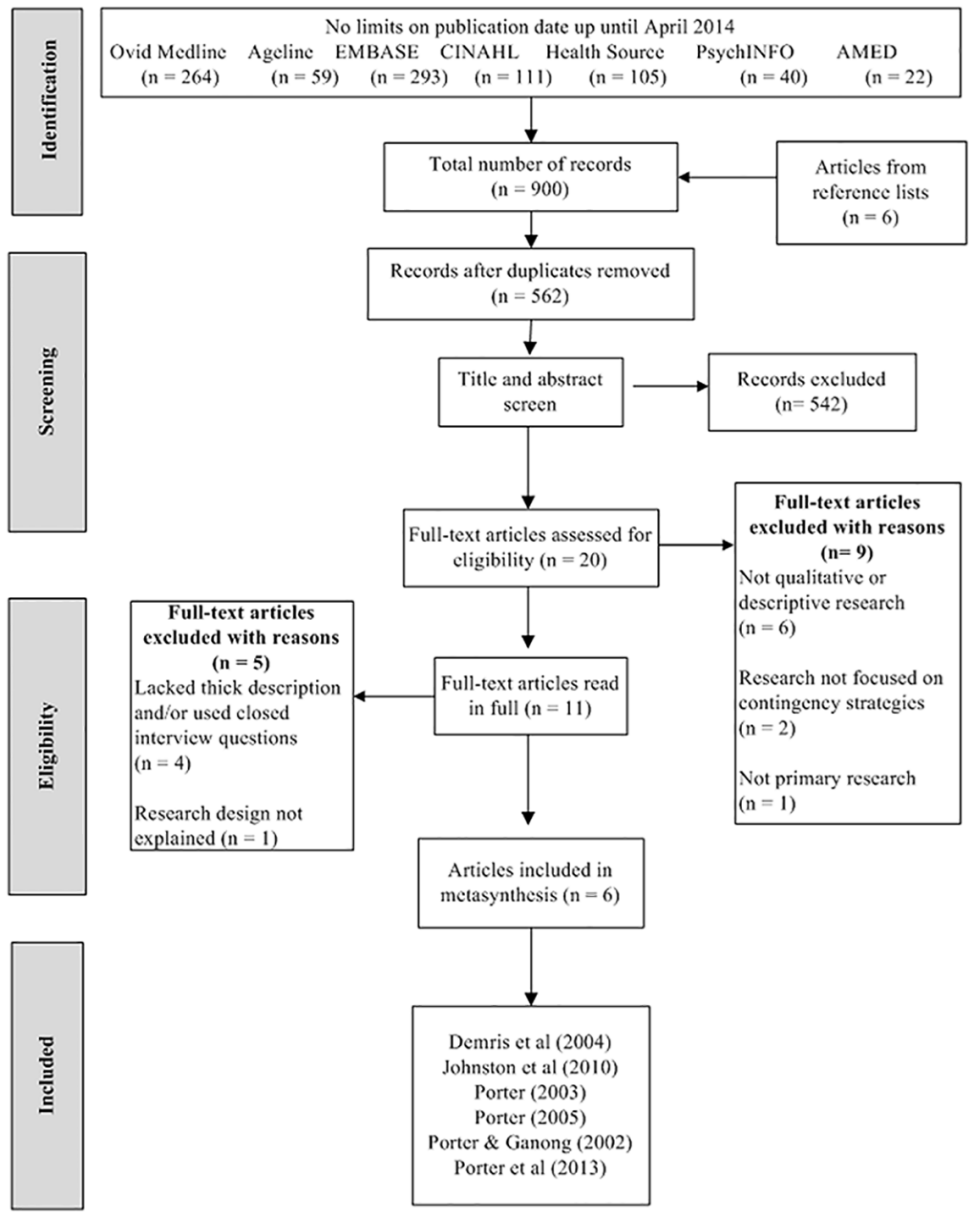
Flow chart of search and screening process.

Initial keyword searching yielded 900 articles including 6 found from reference lists and nothing from grey literature. All duplicate articles were removed and the remaining 562 articles had their titles and abstracts screened against the inclusion/exclusion criteria leaving 20 articles for full text screening against the inclusion and exclusion criteria (KC). This screening resulted in 11 studies for further consideration. Discussion between all authors (KC, CM & SK) about interpretation of the inclusion/ exclusion criteria reduced the included articles to six [23–28]. The search strategy, reasons for study exclusion and outcomes are explained in Fig 1 (PRISMA flow chart). The updated search conducted in February 2016 yielded 38 records for title and abstract screening and subsequently three for full text review [29–31]. Upon review of full text, none of the studies focussed on contingency planning for falls and as such were not considered for inclusion in this systematic review.

### Study description

One of the six papers originated in Australia [24] and the remaining papers originated in the USA. Four American studies by Porter and colleagues formed part of a larger study, which investigated the experiences of older widows using home care. This research used a phenomenological approach and a longitudinal design over 18 months to four years [25–28]. This design used multiple semi structured interviews with participants. The other two studies did not specify a methodological approach; however Demiris, Rantz [23] used focus groups and Johnston, Grimmer-Somers [24] used semi-structured interviews for data collection. Four of the studies focused on PERS [24–27], one study on home monitoring technology [23] and another sought information about any strategies used to reach help quickly [28]. Participants were all recruited through convenience sampling and sample sizes ranged from seven to 40. All studies concentrated on community dwelling older people, except for Demiris, Rantz [23] which also included people living in independent living units, an assisted living facility and a skilled nursing facility. All studies conducted by Porter & colleagues focused only on the perspectives of women ranging from 81–93 years old [25–28]. The remaining studies incorporated both sexes and included participants over 65 years [23, 24]. Further details about the characteristics of the six studies are shown in Table 2.

**Table 2:** Characteristics of the studies included in systematic review.

Author & year	Country of Origin	Focus of Research	Methodology	Sample and Sample Size	Type of contingency planning strategy
Porter, Ganong & Matsuda 2013	USA	Explore the intentions of older homebound women about reaching help quickly- comparing perceptions subscribers and non-subscribers of PERSs	Longitudinal phenomenological design with average of 3 semi-structured interviews over 18 months (interview average 70mins)	Convenience sampling **N = 40** women living alone (21 PERS subscribers/ 19 non PERS subscribers N = 40 women living alone (21 PERS subscribers/ 19 non PERS subscribers) **Age** = ≥ 85 **Health Status** = 95% had broken hip/arthritis or both	Any contingency strategy used to reach help quickly
Porter & Ganong 2002	USA	To explore the experience of frail older women considering whether to be connected to a PERS	Phenomenology Part of a larger longitudinal study- 71 semi structured interviews conducted with 11 women from 1997–2001.	Convenience Sampling **N =** 11 women living alone (6 urban, 5 rural) **Age =** 81–94 (non-PERS owners) **Health Status =** History of falls, frail	PERS
Porter 2005	USA	Explore the nature of older women’s experiences of wearing and using a PERS	Descriptive phenomenology Part of a larger longitudinal study- average of 7 interviews (range 3–10) conducted per woman over 3 years.	Convenience Sampling **N =** 7 women living alone (PERS owners) **Age =** 83–96 years **Health Status =** Self related health of less than excellent, frail, had a health problem which increased likelihood of falls.	PERS
Porter 2003	USA	Explore the experience of older women on having a PERS	Phenomenology Part of a larger longitudinal study: 56 interviews conducted over 3 years (average 7; range 3–10 interviews per woman)	Convenience Sampling **N =** 8 women living alone (4 rural, 4 urban) (had used a PERS at some stage) **Age =** 83–96 years **Health Status =** frail, had a health problem which increased likelihood of falls	PERS
Johnston, Grimmer-Somers & Sutherland, 2010	Australia	Explore the experiences and perceptions of independently living older people who had recently fallen	Qualitative- Semi structured telephone interviews (approximately 20 minutes long)	Convenience Sampling **N =** 31 (26Female, 5 Male) Living in the community, 52% regional SA, 48% urban SA. 64.5% PERS owners **Age =** Over 65 years **Health Status =** All had experienced a fall	PERS
Demris et al 2004	USA	Explore the perceptions and expectations of seniors in regards to “Smart Home” technology installed and operated in their homes with the purpose of monitoring their health status	Qualitative Participants were involved in 1 of 3 focus groups (approximately 1 hour in duration) which were facilitated using a protocol.	Convenience Sampling **N =** 15 (7 Male, 8 Female) living within continuing care retirement facility (this includes independent living units, assisted living facility and skilled nursing facility). Nonusers of this technology **Age =** Over 65 years **Social =** well educated, upper class participants, 97% used computers at home.	Smart Home Technology = videophone and monitoring technology

### Critical appraisal

An understanding about the quality of the research informs reader interpretation of the study findings. However, this appraisal did not result in exclusion of studies or influence analysis of the findings completed by the authors. A summary of the critical appraisal findings against the criteria of credibility, transferability, dependability and confirmability is provided in Table 3. As this was a criterion for inclusion in the meta-synthesis, all studies included a thick description of the findings with adequate supporting quotes that reflected a range of participant views. All studies conducted by Porter demonstrated credibility and transferability through collection of data longitudinally, reflexivity during data collection, multiple methods of triangulation and adequate information about participants, including age mean and range [25–28]. However, the other two studies did not use triangulation, member checking or declare authors preconceptions about the topic and information provided about characteristics of participants was minimal (over 65 years) [23, 24].

**Table 3:** Critical appraisal of studies.

Author	Credibility	Transferability	Dependability	Confirmability
Demiris et al. 2004	X	O	X	X
Johnston et al. 2010	X	O	X	O
Porter et al. 2013	Y	Y	Y	Y
Porter & Ganong, 2002	Y	Y	X	Y
Porter, 2005	Y	Y	O	O
Porter, 2003	Y	Y	O	Y

X = No/Insufficient Information provided to ensure trustworthiness

0 = Some detail and explanation provided but lacking full detail to ensure trustworthiness

Y = Adequate detail and explanation provided to enhance trustworthiness

Evidence of an audit trail or having more than one person involved in reviewing data analysis are both indicators of dependability and these were demonstrated in one study [28]. The other studies lacked transparency in their decision making processes during data analysis or did not state whether a peer had reviewed decisions [23–27]. Three studies used bracketing to declare researcher assumptions along with direct quotes to enhance and confirm findings [26–28]. The remaining studies did not declare any assumptions or provide an audit trail and there were no transparent explanations of how author biases were managed [23–25].

### Findings

From the interpretative analysis of findings from the six studies, three themes were developed about the perspectives of older people about contingency planning for getting help quickly following a fall. These themes were ‘a mix of attitudes’, ‘careful deliberations’ and ‘a source of anxiety’.

#### Theme 1: A mix of attitudes

There was a mix of negative, contemplative and positive attitudes towards the need to have a contingency plan in place for a fall. Those older people who had negative attitudes toward contingency planning were worried around the perceived impact on their identity, including being regarded by others as dependent, old and frail [23, 24]. For some participants having a PERS was associated with feelings of apprehension for the future and a constant reminder that their health was deteriorating [25, 26]. An alarm hanging around their neck that could be seen by others was described as a “*badge of dishonour*” because it symbolised a decline in independence [26]. Those considering getting a PERS were hesitant because they feared the stigma associated with it and were concerned about other people being able to see it under their clothing [26].

Contingency planning for a fall was sometimes regarded as unnecessary by older people. They believed that they were “*getting by fine without it*” [27] and did not need a contingency plan in place to reach help quickly because they believed they were not at risk of having a fall [28]. Some participants had been told by family and health professionals that they should have a PERS [27, 28]. Those participants who subscribed to a PERS because a family member had brought it for them, reported hesitancy about using it and voiced concerns about how useful it was [27], particularly if they preferred to use the phone to reach help [26, 27]. The reasons for preferring to use the phone were not stated.

Some older participants who had previously fallen and were able to independently get up from the floor, placed little value on the establishment and use of a contingency plan and convinced themselves that it may be something that they would look into later, when they believed they really needed it [27, 28]. There were also some participants who used opportunistic help to get them off the floor, such as calling for neighbours or passers-by.

*“My neighbour didn’t hear me*. *I did call out after a while-you feel a bit stupid on the floor you know…..eventually dusk came…..so I thought it was no use, I will try and make myself as comfy as possible as I couldn’t lift myself up to get to the four telephones. I suppose I should get one of these alarms but I don’t fancy one, none of us do”*

[24].

Some people who contemplated the idea of having a PERS reported that they could not afford one [24]. Some participants likened having a PERS to a “lifesaver” [25] and described it as something that made them feel safer and provided peace of mind [24, 25]. Having ‘peace of mind’ was also reported by participants who used home monitoring technology [23].

*“I feel safer, and I feel less afraid too…*..*If I were to press this thing, in a minute or so somebody would say, Mrs W, do you need any help?”*

[25]

#### Theme 2: Careful deliberations

Those who were contemplating developing a contingency plan were thoughtful about what was right for them. Participants that did not own a PERS were not familiar with it and how it worked [25, 28]. They consistently gave reasons why it was not appropriate for them, including the perception that it would be difficult to find an appropriate contact to respond in an emergency, that they couldn’t manage the device due to sensory or cognitive impairment and due to their cost in the United States and Australia. Some existing PERS users were reluctant to wear it due to the perception that they couldn’t use it due to sensory or cognitive impairment [25, 26]. PERS users were also ambivalent about paying monitoring fees [25] and some non-PERS users regarded them as too expensive [24, 25, 27]. Similar financial concerns were raised about the installation of home monitoring technology [23]. Some users justified the cost of the PERS through rationalising their need for it [24] and that they would regret having stopped using or wearing it if they did have an incident where it was needed to alert help [25].

*“Oh Yes*. *Everybody that comes mentions it, not knowing what it entails. But it costs $35 a month, plus a service charge. That’s pretty high”*

[27].

*“It costs me $200 a year for a full service but that is really nothing when you think about it*, *as to what harm could be done so I am quite happy to pay for it”*

[24].

Having “vacant” neighbourhoods or being surrounded by neighbours who may not like to get involved [28] meant that some older people did not have anyone appropriate that they could rely on to contact in an emergency [25, 28]. Participants that did have some contacts to choose from deliberated over who would be most appropriate, their availability to help and whether responders would actually know what to do if they were contacted in an emergency [28].

*“There’s another neighbour*. *She’s kind of moved away; she did live across the street. But I told her I am going to leave you on there. You could at least get some help. She did have a key but she is far enough away now that she didn’t want to keep the key. And if its night I wouldn’t want her to come out and come up here”*

[25].

#### Theme 3: A source of fear and anxiety

The older people were concerned and anxious about using PERS and home monitoring technology. They worried about being unable to use technology in an emergency [23, 26, 28]. In particular, some participants saw sensory and functional restrictions such as vision loss, hearing impairment, loss of tactile sense, loss of balance, difficulty in reading fine print, difficulty in using buttons and cognitive impairments as potential barriers to using technology [23]. These concerns were supported by one participant in Porter (26) who had extremely poor vision and reported difficulties in finding small objects including her PERS and one participant in Johnston, Grimmer-Somers (24) who owned a PERS but could not describe how to operate it. Other participants were concerned that the actual fall may stop them from reaching help quickly [26, 28].

*“If I am by myself I am more or less afraid*. *Of course, I’ve got my Heartline [personal alarm]. ….But something could happen to me, and I couldn’t get to the Heartline too, ‘cause I keep it around my neck. If I’d be unconscious or something, I couldn’t do that”*

[26].

Participants also feared being startled if the PERS was accidentally set off [25, 26] and worried that they would be a burden to others in the event of an accidental activation. A participant explained that the PERS seemed to have “*a mind of its own”* because it would summon for help without the button being pressed [26]. This concern was associated with older women feeling startled by an emergency response centre person or a first responder speaking over the phone, arriving unexpectedly, or breaking into their homes to check on them [25, 26]. Having cameras in participants homes with home monitoring technology was disconcerting for participants, who worried about violation of privacy [23]. However, findings from this same study suggest that anonymising the cameras, so that only shadows and movements could be seen would be more appropriate [23].

*“Just the other night it came on accidentally … Oh boy*, *here comes that booming voice out, ‘Mrs H!”*

[26]

*“I was sittin’ there on the bed, and I heard a car*. *I said, now who’s coming in this time of the night? And he hit that back door and come in. Mama Mama, what’s wrong? I said ‘what do you mean?’ He said ‘they just called me from lifeline and told me to come and see you.’ Some way or other I pressed that button”*

[25].

The possibility of participants being burdensome to others also caused anxiety for some participants [23, 25, 28]. Women reported that the risk of accidental activation and the subsequent alerting of family and friends deterred them from wearing their PERS [26] as they “*don’t like to bother people”* [28], and raised concerns about being taken to hospital and not being able to return home again [24].

## Discussion

This meta-synthesis brought together the findings of qualitative studies with thick descriptions about the perspectives of older people for contingency planning to get help quickly in the event of a fall. Given the nature of the research available, the findings of this meta-synthesis focused on using PERS and home monitoring technology as strategies for contingency planning for a fall. The research found that the perspectives of older people were on a continuum between regarding PERs and home monitoring technology as necessary, contemplating acquiring them in the future and not regarding them as necessary. These perspectives were influenced by a desire to maintain their independence, maintaining their self-identity, worrying about cost and worrying about accidentally activating technology.

Although best practice guidelines have a strong emphasis on falls prevention, some attention is given to preparation about what to do should a fall occur, including implementation of strategies such as PERS [2]. These recommendations encourage older people to value the importance of contingency planning for the possibility of a fall and to prevent the health complications associated with lying on the floor after a fall. However, findings from this review and other literature [32–34] suggests that it cannot be assumed older people and health professionals value contingency planning in the same ways. Differences in values between health professionals and older people can be seen in research about older people’s responses to advice given during falls prevention education [35] and is illustrated by the consistent finding in research that many older people who have a PERS do not activate it if they fall [3, 10, 11].

It is possible that health professionals and family members underestimate the level of anxiety having and using PERS can evoke for older people. Wanting to be independent and worry about admission to hospital was evident in the ‘sources of anxiety’ theme in this meta-synthesis and provides some reasons for not wearing or using PERS [10]. To have a full understanding of how the older people feel about contingency plan options available to them and to ensure that chosen options are successful, it is necessary to consider the preferences and concerns of older people [2]. To support this recommendation, a qualitative thematic synthesis exploring older peoples’ experiences of social care from 30 studies found that involving them in decisions about their care was an essential part of service provision [36].

In keeping with the recommendation to involve older people in decision-making, Fallis, Silverthorne [37] have suggested that health professionals provide detailed information and hands on practice sessions on any contingency plans that involve use of technology. It has been established that older people are less likely to use technology if they are unfamiliar with how to use it and are worried about using it [38]. Similarly, this meta-synthesis found that a lack of knowledge about PERS generated anxiety for older people and was a source of hesitation about deciding to have a PERS or to use it in the event of a fall. By taking the time to fully explain and practice how to use PERS, the anxieties of older people may be partially addressed and this approach may assist in overcoming their perception that they do not have the ability to operate a PERS.

This meta-synthesis found a mix of attitudes and careful deliberations taking place in deciding what to do about contingency planning for a fall. Older people who didn’t believe they were at risk of falling did not see the need for a contingency plan. However, an inability to accurately understand their risk of falling [15] or to disassociate falling from their identity [33] can generate problems for undertaking contingency planning with older people. Zimmerman, Olsen [39] theorised that people in denial about their risk of falling may not realise that advice from health professionals applies to them. As a consequence, it is recommended that health professionals develop positive relationships with older people [36, 39], complete a falls risk assessment and provide information that will support them to understand their potential risk of falling.

Cost was another limitation to putting contingency plans for falls in place. Given that 77% of Australians over 65 receive a form of government pension or allowance, and most pensioners do not have substantial savings or assets, cost is highly relevant for contingency planning [40]. Less expensive options than technology may include linking people with community supports, offering regular check visits or daily phone calls, exploring suitability for portable or mobile phones or for using social networks and neighbour/family for monitoring safety.

### Implications for practice

This research about contingency planning addresses a largely neglected aspect of falls management [14] Best practice guidelines present information and evidence about strategies for falls prevention but options given to health professionals for getting help quickly after a fall are limited [2]. The findings of this meta-synthesis reveal that whilst family members and health professionals may have good intentions in recommending and implementing contingency plans for an older person to get help quickly in the event of a fall (i.e. PERS), these plans may not be valued or implemented if the older person is not actively involved in the decision making. In fact, the contingency plan may evoke anxiety and a negative response for the older person.

Actively involving the older person in decision making includes exploring and being directed by their perceptions and preferences rather than taking a ‘one size fits all’ approach [36]. There appears to be a practice of health professionals reacting to falls risk by suggesting contingency plans that involve technology (i.e. PERS) without fully considering all of the options for getting help quickly. It is possible that solutions that are ‘low-tech’ may be just as effective and more acceptable to the older person. Based on the imperative to avoid the long-lie on the floor, we recommend that urgent attention be given to development of guidelines for contingency planning for falls.

### Strengths and limitations

Literature published about the perspectives of older people toward contingency planning for a fall is minimal. As a consequence, the number of studies included in the meta-synthesis was small; however, this is appropriate for a meta-synthesis when the review question and inclusion criteria are specific (see [41]). Also, four of the six studies included in this meta-synthesis were published by the same author. Whilst this could be regarded as a limitation, it is not unusual in meta-syntheses to extract and translate into one another findings from a small number of studies from the same health research team (see [42]).

Four studies formed part of a large longitudinal study involving older women in the United States between 81 and 93 years of age [25–28]. As a result, the transferability of these findings to men and to older people between 65 and 81 is limited. In addition, four of the included studies focused on PERS [24–27] and there was limited literature about other contingency planning options. Advancements in technology since these studies were conducted may mean there are now friendlier and easier to manage contingency planning options available to older people that are not captured in this meta-synthesis. All authors were involved in key stages of the review which contributed to the rigour of the findings. An audit trail was kept of all analytical decisions made [19]. Including studies with thick description enhanced the ability of the authors to lift the findings of the meta-synthesis to a higher order of interpretation in keeping with the methodology [21, 22].

### Recommendations for future research

To further develop the findings of this meta-synthesis, research about the effectiveness of PERS and home monitoring technology is recommended as well as exploring other forms of contingency planning that both are both technological and non-technological. It is also recommended that further feedback be sought from older people about their perceptions of services provided that discuss contingency planning for falls and to seek their suggestions about ways to promote choice and consideration of their preferences and concerns. This is particularly relevant in the current health care context with the emergence of client-centred and patient-directed care, where the older person is at the centre of care and is an active participant in decision making, More insights from the older people who consume services may contribute to knowledge about why contingency plans for falls are sometimes not enacted when they are required.

## Conclusion

This meta-synthesis, the first of its kind in the field of contingency planning for older people for falls, has highlighted that plans may not be valued by older people in the same way that health professionals do. Desirable contingency plans vary between people and are often influenced by a number of factors including lack of awareness of and knowledge about specific strategies, and different attitudes, fears and worries. Without recognising these factors, any implementation is likely to fail. Therefore it is important for health professionals to consider older peoples’ circumstances and provide choices in decision making so that targeted strategies for contingency planning for falls can be implemented.

## Supporting information

S1 PRISMA Checklist (DOC)
